# Directed evolution of genetically encoded LYTACs for cell-mediated delivery

**DOI:** 10.1073/pnas.2320053121

**Published:** 2024-03-22

**Authors:** Jonathan Lee Yang, Sean A. Yamada-Hunter, Louai Labanieh, Elena Sotillo, Joleen S. Cheah, David S. Roberts, Crystal L. Mackall, Carolyn R. Bertozzi, Alice Y. Ting

**Affiliations:** ^a^Department of Chemistry, Stanford University, Stanford, CA 94305; ^b^Sarafan ChEM-H, Stanford University, Stanford, CA 94305; ^c^Center for Cancer Cell Therapy, Stanford Cancer Institute, Stanford University School of Medicine, Stanford, CA 94305; ^d^Department of Bioengineering, Stanford University, Stanford, CA 94305; ^e^Parker Institute for Cancer Immunotherapy, San Francisco, CA 94305; ^f^Department of Biology, Stanford University, Stanford, CA 94305; ^g^Department of Pediatrics, Stanford University School of Medicine, Stanford, CA 94305; ^h^Department of Medicine, Stanford University School of Medicine, Stanford, CA 94305; ^i^HHMI, Stanford University, Stanford, CA 94305; ^j^Department of Genetics, Stanford University, Stanford, CA 94305; ^k^Chan Zuckerberg Biohub-San Francisco, San Francisco, CA 94158

**Keywords:** targeted protein degradation, cell therapy, protein engineering

## Abstract

Lysosome-targeting chimeras (LYTACs) are a promising therapeutic modality to drive the degradation of extracellular proteins. However, early versions of LYTAC contain synthetic glycopeptides that cannot be genetically encoded. Here, we present our designs for a fully genetically encodable LYTAC (GELYTAC), making our tool compatible with integration into therapeutic cells for targeted delivery at diseased sites. To achieve this, we replaced the glycopeptide portion of LYTACs with the protein insulin-like growth factor 2 (IGF2). After showing initial efficacy with wild-type IGF2, we increased the potency of GELYTAC using directed evolution. Subsequently, we demonstrated that our engineered GELYTAC construct not only secretes from HEK293T cells but also from human primary T-cells to drive the uptake of various targets into receiver cells. Immune cells engineered to secrete GELYTAC thus represent a promising avenue for spatially selective targeted protein degradation.

## Significance

Better therapeutic windows can be achieved by targeting therapeutics to their desired sites of action. For protein therapeutics, this might be achieved by engineering cell therapies that home to a tissue of interest and secrete the biologic drug locally. Here, we demonstrate that human primary T cells can be engineered to produce genetically encoded lysosome-targeting chimeras (GELYTACs). These GELYTACs mediate the degradation of extracellular proteins associated with cancer progression. Thus, cells engineered to produce GELYTACs represent an encouraging potential direction for cancer therapeutics.

**Fig. 1.**
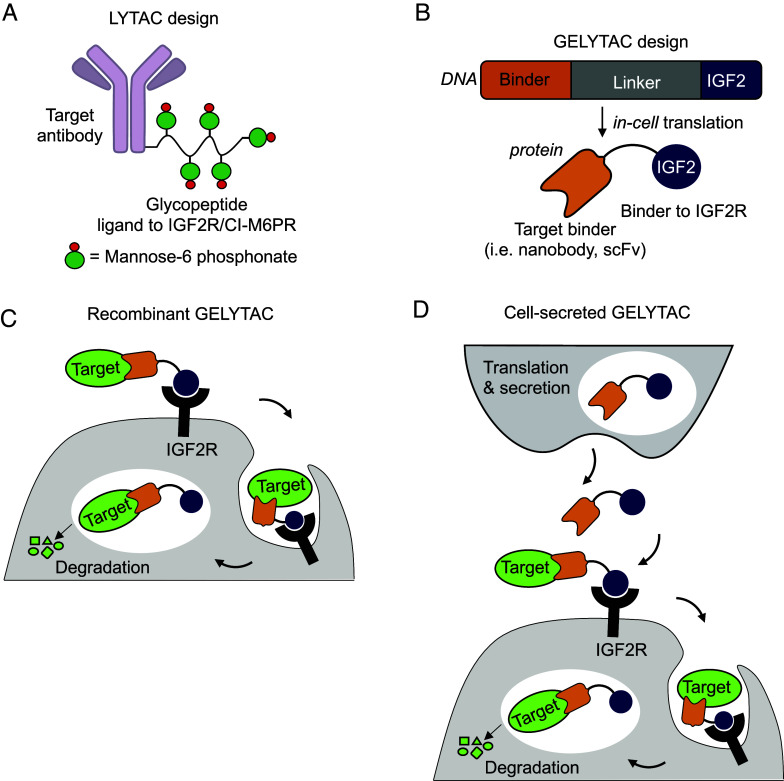
Design and applications of GELYTAC. (*A*) Design of LYTAC (2), in which the binder to IGF2R/M6PR is a synthetic glycopeptide bearing mannose-6 phosphonate groups. LYTAC is not genetically encodable. (*B*) Genetically Encoded LYTACs (GELYTACs) are bifunctional proteins consisting of a binder (i.e., nanobody or scFv) to the target of interest and IGF2, a 7.5-kDa protein that binds to IGF2R. (*C*) GELYTACs can be utilized as a recombinant protein, where the target is internalized via GELYTAC binding to IGF2R and then degraded in the lysosome. (*D*) Alternatively, GELYTACs can be secreted by cells (such as therapeutic T cells) to act on local targets.

Lysosome-targeted degradation is an emerging therapeutic modality that facilitates the degradation of membrane and soluble extracellular proteins. Compared to traditional therapeutic modalities, such as small molecule or antibody-based inhibitors, targeted protein degradation offers increased potential potency and broadens the druggable proteome (1). The first generation of this technology came in the form of lysosome-targeting chimeras (LYTACs), which are bifunctional molecules comprised of an antibody that binds to a cell surface or secreted protein of interest (POI) conjugated to a ligand that binds a lysosome trafficking receptor such as the insulin growth factor 2 receptor (IGF2R, also known as CI-M6PR) (Fig. 1*A*) (2–4). Since then, other technologies have been developed, such as AbTACs (5), ProTABs (6), and KineTACs (7), among others (8–22). These use similar bifunctional molecules to recruit POIs either to lysosome trafficking receptors or plasma membrane-associated ubiquitin ligases and have opened a promising direction in the field of targeted protein degradation.

There is increasing interest in new technologies that target therapeutic molecules to the sites of their desired action (23). This has recently been accomplished by encoding the production of therapeutic proteins in the genomes of targeted cell therapies. For example, therapeutic T cells or natural killer (NK) cells have been engineered to secrete scFvs (24), bispecific T cell engagers (25, 26), or cytokines (27–30) within tumor microenvironments, thereby concentrating the activity of these proteins at the desired tissue site. We are interested in developing a version of LYTACs that can be similarly delivered.

The idea of spatially selective targeted degraders is complementary to cell-type selective LYTACs (7). However, the identification of cell-type selective lysosomal trafficking receptors remains a work in progress. Furthermore, disease states often involve multiple cell types (e.g., the tumor microenvironment which comprises a heterogeneous mixture of tumor cells, immune cells, and bystander cells). Therefore, an ideal and more effective approach should drive the degradation of the target protein by all cell types in the TME, not just a single one.

Here, we report the development of Genetically Encoded LYTACs, which we term GELYTACs. These are encoded by a single transgene that can introduced into therapeutically relevant cells. The GELYTAC is comprised of two small protein modules: A small protein binder (i.e., nanobody or scFv) to a target of interest and an evolved variant of IGF2 (insulin growth factor 2) that binds to IGF2R. We improved the IGF2 scaffold via directed evolution and demonstrated its ability to selectively target extracellular mCherry, TGF-β, and shed IL6R ectodomain. To illustrate the potential utility for cell-based therapeutics, we also show that engineered primary human T cells secreting GELYTACs can effectively induce uptake of aforementioned targets by tumor cells. Our study introduces a promising format for the effective and versatile clearing of extracellular proteins by degraders secreted by engineered cells. This could potentially be later applied for the spatially selective degradation of targets in the tumor microenvironment.

## Results

### Design of GELYTACs.

To design an all-protein, genetically encodable bifunctional molecule for targeted degradation of extracellular proteins, we fused a small protein binder via a flexible linker to IGF2 peptide (Fig. 1*B*). IGF2 binds to domain 11 of IGF2R with a K_D_ of 4.5 nM (31) and is subsequently shuttled to the lysosome (32, 33). This biologic can either be administered recombinantly or be delivered by therapeutic cells as a cell therapy (Fig. 1 *C* and *D*).

**Fig. 2.**
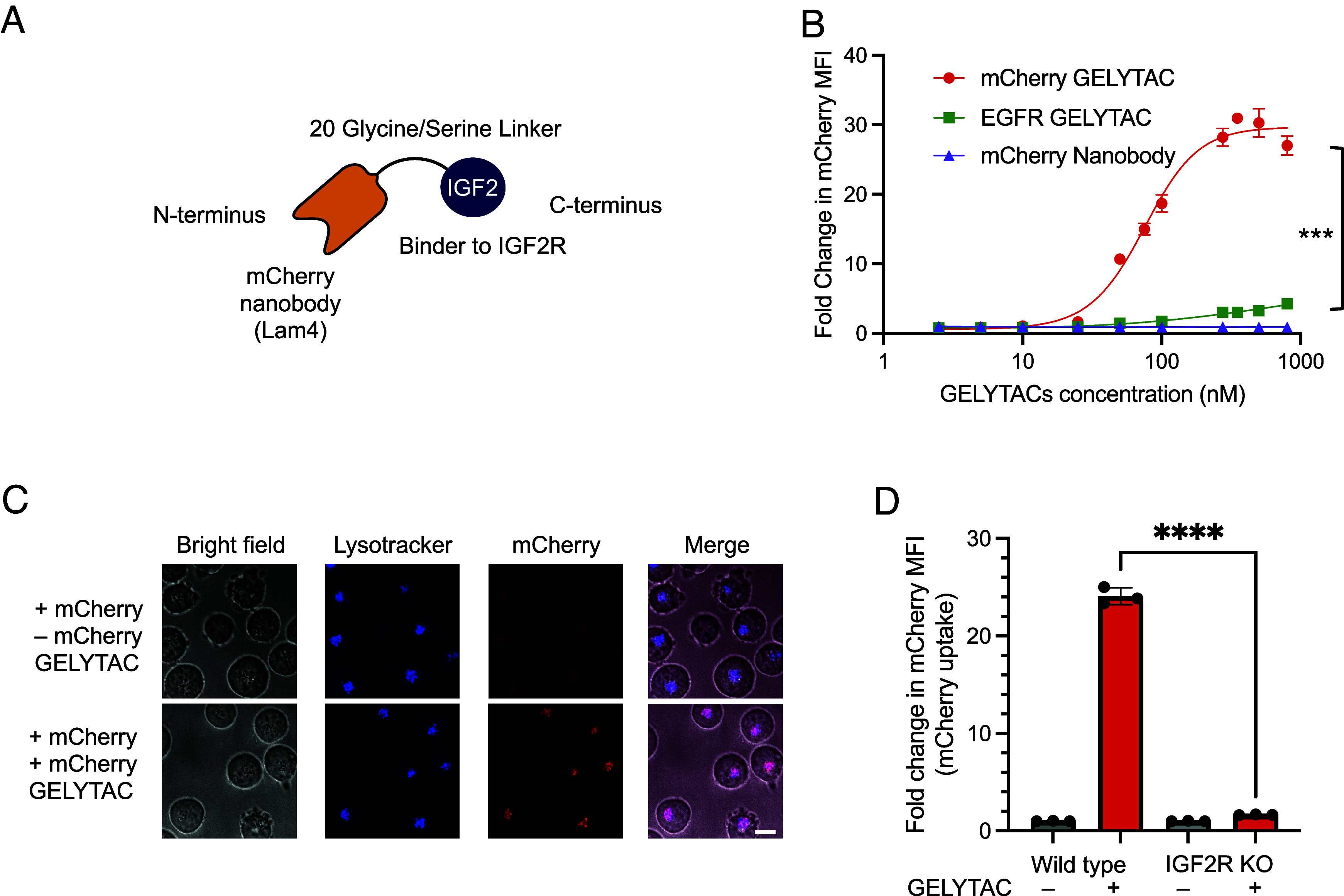
Recombinant GELYTACs mediate internalization of mCherry by K562 cells. (*A*) Design of mCherry GELYTAC. LaM4 is a nanobody that binds to mCherry with a K_D_ of 180 pM (34). (*B*) mCherry internalization as a function of GELYTAC concentration. Median fluorescence intensity (MFI) of K562 cells was measured 24 h after treatment with mCherry GELYTAC (circles) and recombinant mCherry (100 nM). Negative controls are mCherry nanobody only (triangles) and a GELYTAC targeting EGFR (squares). Errors bars represent the SD from three biological replicates. *** = *P* < 0.001 (determined using parametric *t* test). (*C*) Confocal fluorescence images of live K562 cells after 1-h treatment with 100 nM mCherry and 275 nM mCherry GELYTAC. (Scale bars, 10 μm.) (*D*) GELYTAC-mediated mCherry internalization by wild-type and IGF2R KO K562 cells. The experiment was performed at 275 nM of GELYTACs and 100 nM mCherry for 4 h. Errors bars represent the SD from three biological replicates. **** = *P* < 0.0001 (determined using parametric *t* test).

As a proof of concept, we designed and generated GELYTAC targeting a model protein, mCherry. We selected the nanobody LAM4 which binds to mCherry with a K_D_ of 180 pM (34) and fused it to IGF2 (Fig. 2*A*). We were able to easily produce mCherry GELYTAC by recombinant expression in *Escherichia coli* (*SI Appendix*, Fig. S1*A*). To test mCherry GELYTAC’s ability to selectively target and mediate internalization of mCherry into cells, we treated K562 leukemia cells with a mixture of mCherry protein (100 nM) and mCherry GELYTAC (from 0 to 1,000 nM) for 24 h (Fig. 2*B*). Since mCherry is more resistant to lysosomal degradation (35), we expect GELYTAC to drive fluorescent mCherry accumulation into cells’ lysosomes. Flow cytometry showed a 30-fold increase in mCherry fluorescence in K562 cells at 350 nM of mCherry GELYTAC while controls with mCherry nanobody only or an alternate-targeting GELYTAC (EGFR GELYTAC) showed minimal mCherry uptake in K562 cells across all concentrations. In agreement with the flow cytometry data, fluorescence microscopy showed that mCherry colocalized with stained lysosomes, in cells treated with mCherry GELYTAC (Fig. 2*C* and *SI Appendix*, Fig. S1*C*). A time course analysis shows that mCherry uptake continues at 48 h of mCherry GELYTAC treatment (*SI Appendix*, Fig. S1*B*).

To elucidate the mechanism of action, we repeated the flow cytometry experiments using IGF2R knockout K562 cells. In these samples, no uptake of mCherry was observed (Fig. 2*D*) in the presence of mCherry GELYTAC. These data suggest that GELYTAC effectively targets soluble extracellular proteins for lysosomal targeting via recruitment of IGF2R.

### Directed Evolution of GELYTACs.

We designed GELYTAC such that they can be secreted by therapeutic cells. However, unlike recombinant proteins, for which we can specify dosing concentrations, we cannot control secreted GELYTAC concentrations. Thus, we aimed to make GELYTACs more effective at lower concentrations by lowering their EC_50_ through directed evolution, which is facilitated by the genetically encoded nature of GELYTAC.

**Fig. 3.**
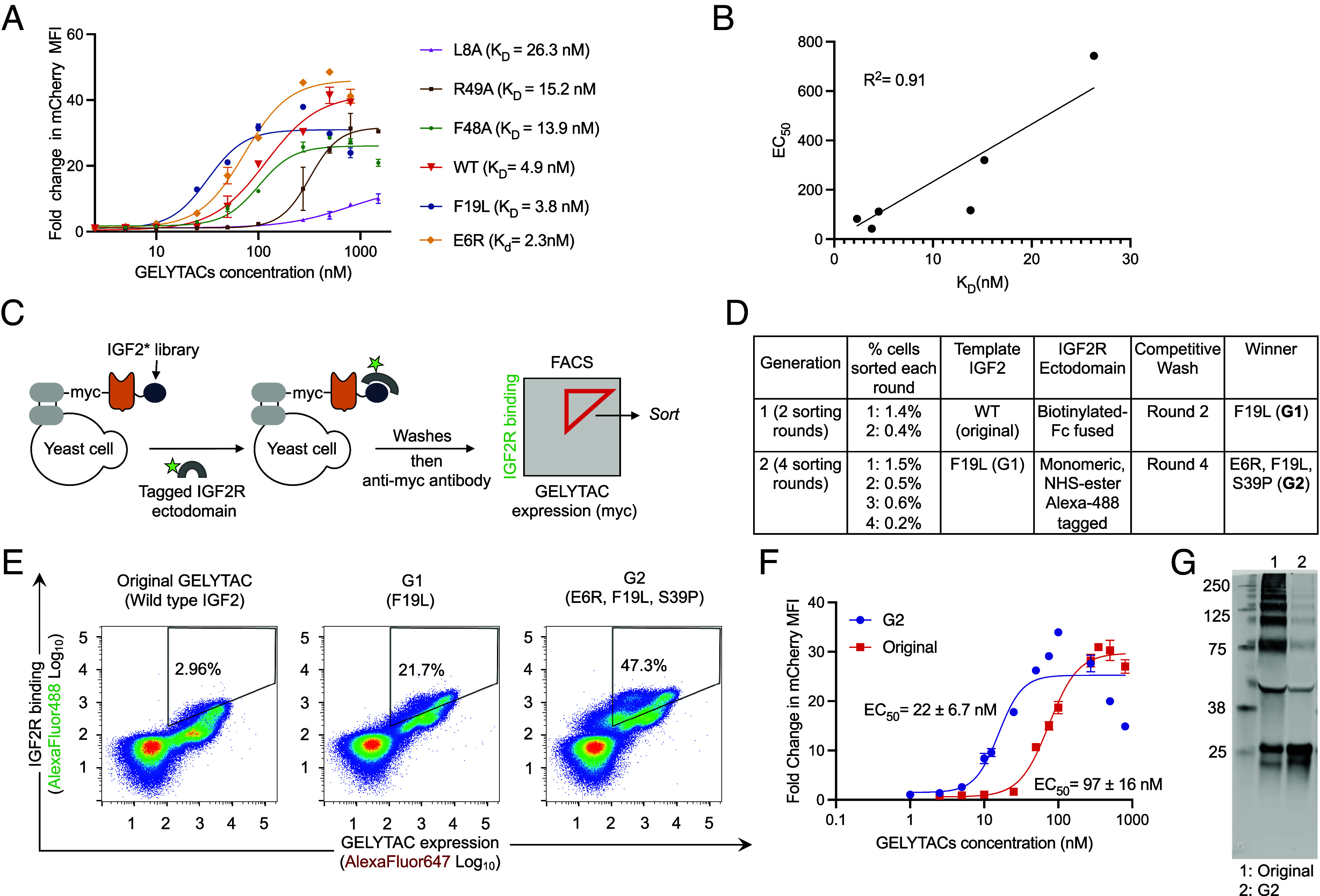
Directed evolution of GELYTAC. (*A*) The effect of point mutations in IGF2 on GELYTAC-mediated internalization of mCherry. mCherry (100 nM) uptake assay was performed as in Fig. 2*B*. Errors bars represent the SD from three biological replicates. (*B*) EC_50_ calculated from the data in Fig. 3*A* plotted against published K_d_ values (31, 36). (*C*) Directed evolution scheme. mCherry GELYTAC library is displayed as a fusion to Aga2p. Yeast cells were treated with AlexaFlour488-labeled IGF2R ectodomain (100 nM), washed three times, then stained with anti-myc antibody and sorted by FACS. (*D*) Summary of selection conditions used across two generations and six rounds of sorting. (*E*) 2D flow cytometry analysis of yeast expressing original and evolved mCherry GELYTACs (G1 and G2). Cells were stained with AF-488 tagged-monomeric IGF2R ectodomain and anti-myc antibody as in Fig. 3*C*, then washed and analyzed by flow cytometry. (*F*) GELYTAC-mediated internalization (original and G2) of mCherry (100 nM) by K562 cells, performed as in Fig. 2*B*. Errors bars represent the SD from three biological replicates. EC_50_ values are averaged over three sets of dose–response curves. Errors in EC_50_ represented as SD. (*G*) Anti-FLAG western blot of original and G2 mCherry GELYTACs secreted from HEK293T cells. SDS-PAGE was run under nonreducing conditions to reveal oligomerization of original GELYTAC and reduced oligomerization of G2 GELYTAC.

We hypothesized that by evolving the IGF2 peptide in GELYTAC, we would be able to achieve tighter binding to IGF2R and consequently more potent POI (protein of interest) internalization and degradation. First, to test whether higher affinity of IGF2 translates into a more efficacious GELYTAC, we created mCherry GELYTACs using a series of known point mutants of IGF2 with varying affinities for IGF2R (Fig. 3*A*) (31, 36). We observed a negative association between IGF2 variants’ K_D_’s and mCherry internalization by the corresponding mCherry GELYTAC (Fig. 3*B*). This provided the rationale to engineer an improved IGF2 binder for improving GELYTAC potency through yeast surface display directed evolution.

Using the mCherry GELYTAC as a scaffold, we introduced diversity in the IGF2 domain by error-prone PCR. The library was displayed on the yeast cell surface via fusion to the C terminus of the yeast mating protein Aga2p (37–40). To select for clones capable of binding to IGF2R with high affinity, we incubated the yeast library with tagged recombinant IGF2R (Fc-fused or monomeric). Staining with anti-myc antibody was used to quantify the GELYTAC expression level. Two-dimensional fluorescence-activated cell sorting (FACS) sorting was used to enrich library members with a high IGF2R/anti-myc intensity ratio (Fig. 3*C*). To increase selection stringency in the last rounds, we modified our staining protocols to either include competitive washes or eliminate the avidity effects from Fc-fused IGF2R by shifting to monomeric IGF2R (Fig. 3*D*). Ultimately, after 6 rounds of sorting over 2 generations, we isolated a clone with the IGF2 mutations E6R, F19L, and S39P (termed “G2”). G2 exhibited improved binding to IGF2R’s ectodomain at expression levels matched to that of wild-type IGF2 (Fig. 3*E* and *SI Appendix*, Fig. S2 *A–C*). This mutant contains two published mutations (31, 36) known to lower IGF2’s K_D_ (E6R and F19L), as well as a novel mutation, S39P. We verified that S39P is a crucial mutation for G2’s improved binding by comparing G2’s triple mutations to the E6R and F19L double mutant (*SI Appendix*, Fig. S2*E*).

To verify that the benefits of directed evolution translate into a cellular context, we compared the efficacy of the original GELYTAC to our evolved G2 GELYTAC in mCherry uptake by K562 cells and saw dramatic reduction of the EC_50_ from 97 nM to 22 nM (Fig. 3*F*). Finally, to see whether both the original and G2 GELYTACs can be secreted by HEK293T cells, we ran an anti-FLAG western blot of the supernatant of HEK293T cells transfected with these constructs. Not only did both constructs secrete well, the evolved G2 GELYTAC also exhibited decreased propensity to oligomerize (Fig. 3*G* and *SI Appendix*, Fig. S3 *C* and *F*). This increases the amount of biologically active material, which is likely to contribute to improvements in efficacy of downstream cell-based therapy applications where GELYTACs cannot be purified.

### Cell-Based Secretion of GELYTACs and Knockdown of Other Targets.

**Fig. 4.**
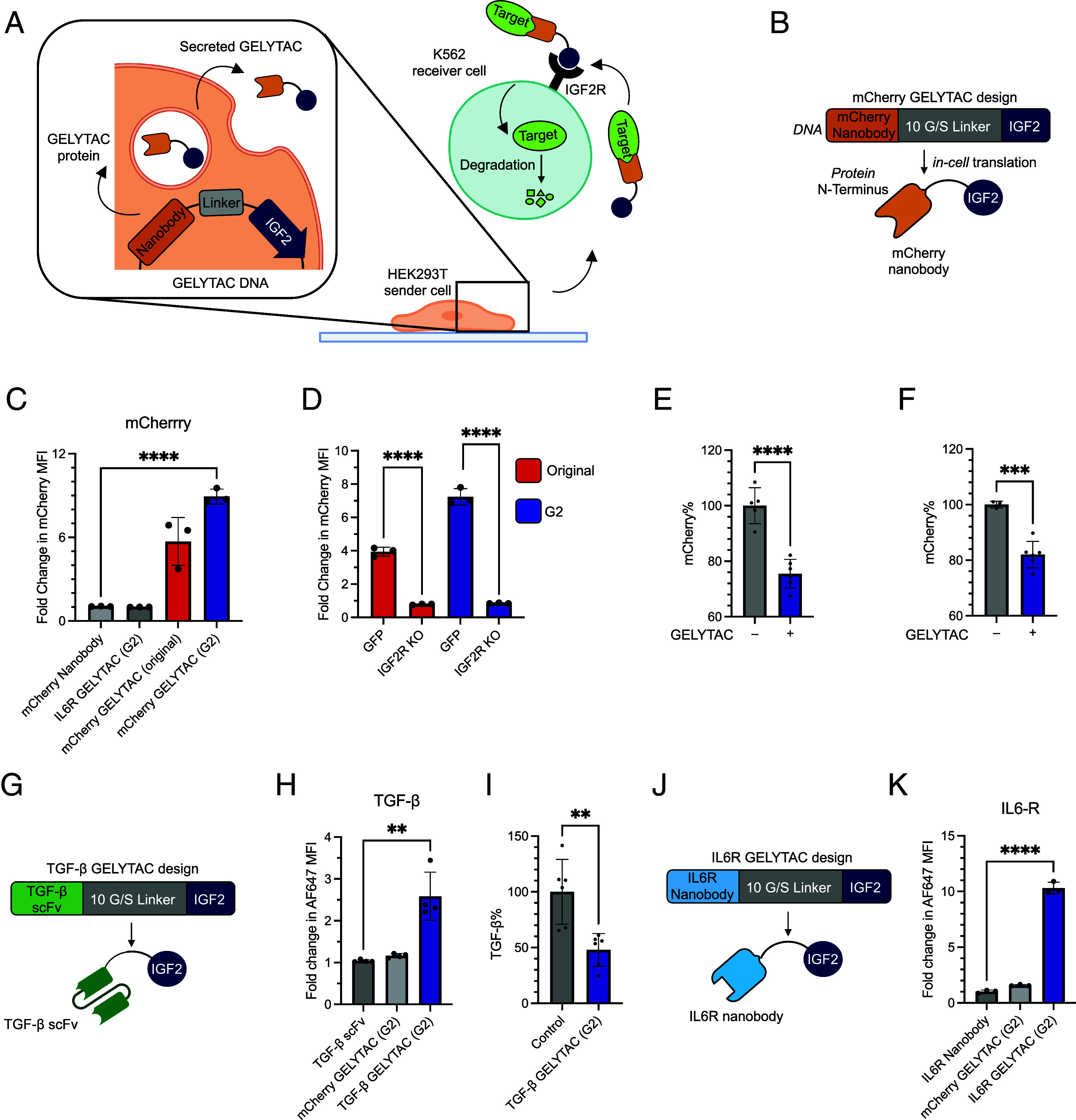
Delivery of GELYTAC via cell secretion. (*A*) Schematic of cocultured sender and receiver cells. Adherent HEK293T cells secrete GELYTAC that acts on suspended K562 cells to internalize and degrade targets. (*B*) Design of mCherry GELYTAC used in coculture experiments. (*C*) Cocultured cells were treated with mCherry (100 nM) for 24 h. Then, K562 receiver cells were separated and analyzed by flow cytometry. HEK293T secreting either original or G2 GELYTAC were tested. Control GELYTAC targets IL6R instead of mCherry. The measured concentration of mCherry GELYTAC (original), mCherry GELYTAC (G2), mCherry nanobody, and IL6R control GELYTAC (G2) at 24 h are 48 ± 5 nM, 78 ± 9.9 nM, 2.0 ± 0.4 nM, and 33 ± 3.4 nM (*SI Appendix*, Figs. S3*B* and S6 *A–D*). Errors bars represent the SD from three biological replicates. **** = *P* < 0.0001 (determined using parametric *t* test). (*D*) Cocultures comprising either wild-type or IGF2R KO K562 cells were treated with mCherry (100 nM) for 24 h and analyzed as in Fig. 4*C*. (*E*) To quantify mCherry (10 nM) clearance in the coculture system, media was analyzed for mCherry fluorescence 72 h after mCherry addition using a plate reader. This experiment was repeated five times. Errors bars represent the SD from five biological replicates. **** = *P* < 0.0001 (determined using parametric *t* test). (*F*) To quantify mCherry (10 nM) degradation, both cells and media were collected and analyzed for mCherry fluorescence after mCherry addition using a plate reader. This experiment was repeated five times. Errors bars represent the SD from five biological replicates. *** = *P* < 0.001 (determined using parametric *t* test). (*G*) Design of TGF-β GELYTAC utilizing a TGF-β scFv derived from the clinical candidate Fresolimumab (41). (*H*) Cocultured cells were treated with AlexaFluor-647 tagged TGF-β (100 nM) for 24 h. Then, K562 receiver cells were separated and analyzed by flow cytometry. HEK293T cells secreting G2 GELYTAC were tested. Control GELYTAC targets mCherry instead of TGF-β. The measured concentration of TGF-β GELYTAC (G2), TGF-β scFv, and mCherry control GELYTAC (G2) at 24 h are 450 ± 40 pM, 110 ± 20 pM, and 78 ± 9.9 nM (*SI Appendix*, Figs. S3*D* and S6 *E–H*). Errors bars represent the SD from three biological replicates. ** = *P* < 0.01 (determined using parametric *t* test). (*I*) To quantify degradation of TGF-β, after addition of 1 nM biotinylated TGF-β for 72 h, both cells and media were collected and analyzed for biotin signal via streptavidin-800 blot. This experiment was repeated six times. The control is HEK293T cells co-transfected with both TGF-β scFv and mCherry GELYTAC (G2). Errors bars represent the SD from six biological replicates. ** = *P* < 0.01 (determined using parametric *t* test). (*J*) Design of IL6R GELYTAC utilizing an IL6R nanobody derived from clinical candidate ALX-0061 (42). (*K*) Cocultured cells were treated with AlexaFluor-647 tagged IL6R (100 nM) for 24 h, then K562 receiver cells were separated and analyzed by flow cytometry. HEK293T cells secreting G2 GELYTAC were tested. Control GELYTAC targets mCherry instead of IL6R. The measured concentration of IL6R GELYTAC (G2), IL6R nanobody, and mCherry control GELYTAC (G2) at 24 h are 33 ± 3.4 nM, 40 ± 2.2 nM, and 78 ± 9.9 nM (*SI Appendix*, Figs. S3*G* and S6 *I* and *J*). Errors bars represent the SD from three biological replicates. *** = *P* < 0.001 (determined using parametric *t* test).

Next, we explored an alternate mode of GELYTAC administration: secretion by HEK293T sender cells to drive uptake of soluble targets into receiver K562 cells (Fig. 4*A*). To achieve this, we cocultured HEK293T cells transfected with mCherry GELYTAC (original or G2), control GELYTAC (alternate-targeting, IL6R GELYTAC), or mCherry nanobody only with GFP-expressing K562 cells for 24 h in media supplemented with 100 nM mCherry (Fig. 4*B*). We verified the presence of all constructs in the conditioned media at 24 h by western blot and observed comparable secretion among the constructs (*SI Appendix*, Fig. S3 *A* and *B*). We also observed another band of slightly lower mass in both GELYTAC secretion samples. To determine the identity of the second band, we analyzed both GELYTACs by top–down mass spectrometry (MS); while both contained a portion of truncated GELYTAC (C-terminal truncation of 28 and 27 amino acids for original and G2, respectively), the predominant species corresponded to the full length (*SI Appendix*, Fig. S4 *A–D*). This truncation would likely result in decreased binding to IGF2R while maintaining binding to the target protein.

Next, we analyzed the receiver cells using flow cytometry and observed a ~sixfold and ~ninefold increase in mCherry median fluorescence intensity in K562 cells cocultured with HEK293Ts secreting original and G2 mCherry-GELYTACs, respectively (Fig. 4*C*). We then performed the same experiment with the original and G2 mCherry GELYTACs on IGF2R KO K562 cells and saw no apparent uptake by the IGF2R KO cells, demonstrating that uptake of proteins in the coculture model is mediated by IGF2R, as we observed for treatment with the recombinant GELYTAC.

While we had observed robust mCherry uptake into receiver cells as a proxy for protein degradation, to determine whether secreted mCherry GELYTACs could mediate effective clearance and degradation of mCherry, we analyzed mCherry fluorescence in the coculture supernatant for mCherry clearance and the combined supernatant plus cells for mCherry degradation using a plate reader. After incubation of GELYTAC secreting HEK293T cells with K562 receiver cells, we observed that ~25% of mCherry is cleared (Fig. 4*E*) from the media and ~20% of mCherry is degraded (Fig. 4*F*).

Finally, we demonstrate that the GELYTAC design we optimized for targeting mCherry can be generalized to internalization of more therapeutically relevant targets by simply exchanging the nanobody. To this end, we developed GELYTACs targeting soluble proteins TGF-β and IL6R. TGF-β and IL6R are immunosuppressive factors in the tumor microenvironment, so depleting these soluble factors could help improve the efficacy of cancer immunotherapy treatments, such as immune checkpoint blockade and CAR-T therapy (23). Local degradation of these targets is attractive because systemic targeting of immunosuppressive factors has been shown to be toxic (43). We designed the TGF-β GELYTAC by fusing our evolved IGF2 with a TGF-β scFv derived from the clinical candidate antibody Fresolimumab (41) (Fig. 4*G*). We tested the TGF-β GELYTAC in a coculture system with HEK293T secreting the TGF-β GELYTAC, GFP-expressing K562, and media supplemented with 100 nM AlexaFluor647 (AF-647)-tagged TGF-β. Like in the mCherry assays, we observed a ~2.6-fold increase in AF-647 fluorescence in K562 cells (Fig. 4*H* and *SI Appendix*, Fig. S3 *C* and *D*). We also determined whether TGF-β GELYTAC could mediate the degradation of TGF-β. To do this, we added 1 nM of biotinylated TGF-β to K562 cells cocultured with HEK293T cells secreting mCherry GELYTAC plus TGF-β scFv, or TGF-β GELYTAC, and analyzed via streptavidin western blot a mixture of the cell lysate and supernatant. We observed that the TGF-β GELYTAC mediated ~52% reduction in biotinylated TGF-β when compared to the combined mCherry GELYTAC and TGF-β scFv control (Fig. 4*I* and *SI Appendix*, Fig. S3*E*).

To test clearance and degradation of another target, IL6R, we developed a GELYTAC consisting of evolved IGF2 fused to a clinical candidate IL-6R nanobody (ALX-0061) (42) (Fig. 4*J*). In the coculture assay, we supplemented the media with 100 nM AF-647-tagged IL-6R and saw ~10-fold increase in AF-647 signal in the K562 cells when cocultured with IL6R GELYTAC secreting HEK293T cells (Fig. 4*K* and *SI Appendix*, Fig. S3 *F* and *G*).

In these experiments, the MFI increase mediated by secreted TGF-β GELYTAC was lower compared to mCherry and IL6R GELYTACs. This could be due to binding of TGF-β to the TGF-β1 receptor or other receptors resulting in increased background signal. Development of tighter binding nanobodies could potentially improve efficacy of this class of GELYTAC that competes with endogenous cell-surface receptors for target binding. Overall, these results demonstrate the modularity and potential of GELYTAC to recognize a wide range of targets by simply exchanging the POI binder.

**Fig. 5.**
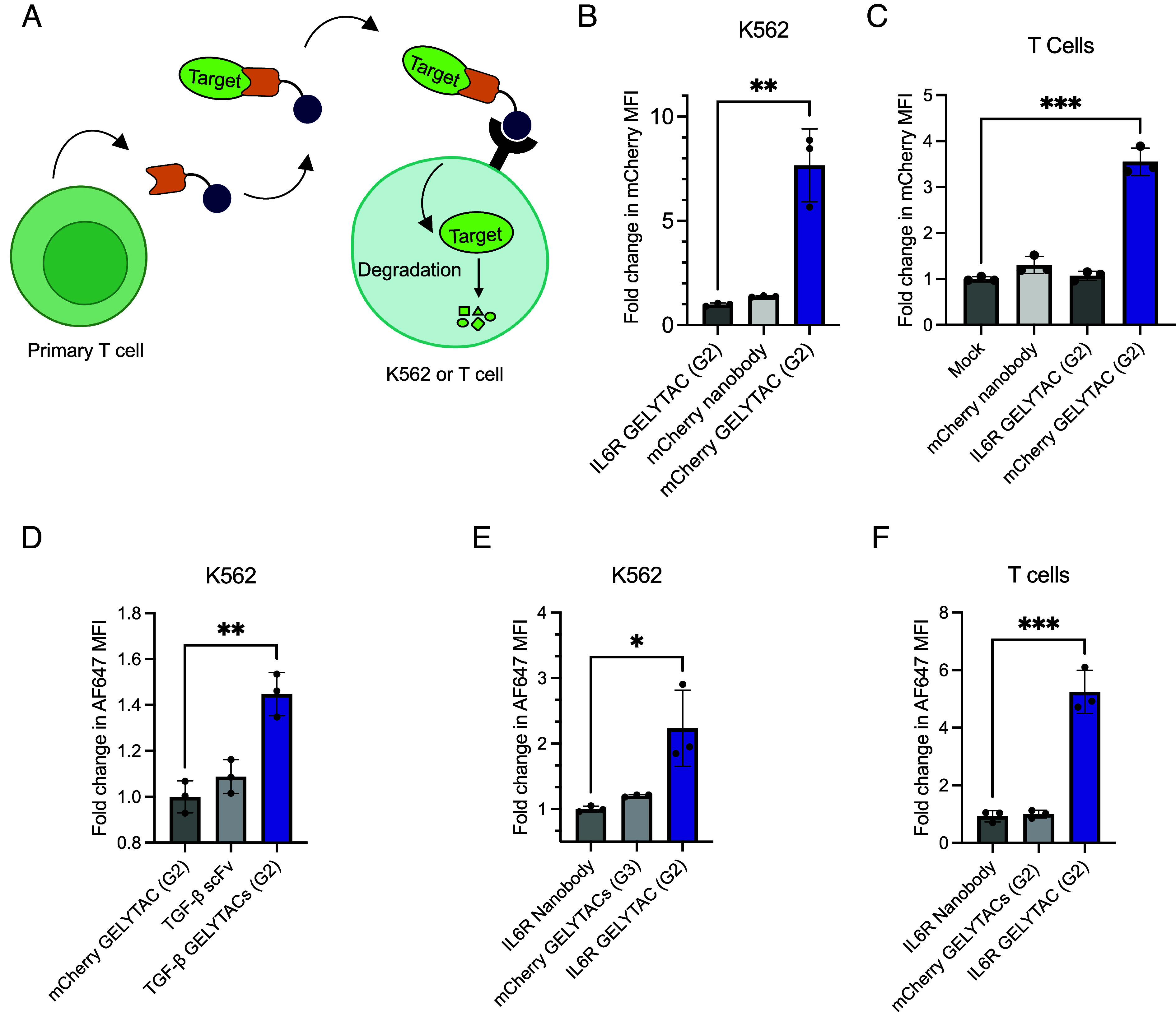
GELYTACs secreted from primary T cells mediate uptake of multiple targets. (*A*) Schematic of T cell coculture system. Suspended human primary T cells secrete GELYTAC that acts on suspended K562 cells and T cells to internalize and degrade targets. (*B*) Cocultured cells were treated with mCherry (100 nM) for 61 h. Then, K562 cells were analyzed by flow cytometry. T cells secreting G2 GELYTAC were tested. Control GELYTAC targets IL6R instead of mCherry. The measured concentration for mCherry GELYTAC (G2), mCherry nanobody, and IL6R control GELYTAC (G2) at 72 h are 32 ± 4.3 nM, 190 ± 32 nM, and 13 ± 0.73 (*SI Appendix*, Figs. S5*B* and S7 *A* and *B*). Errors bars represent the SD from three biological replicates. *** = *P* < 0.001 (determined using parametric *t* test). (*C*) Experiment performed as in (*B*) but measuring fold change in mCherry MFI in human primary T cells from the coculture system. Errors bars represent the SD from three biological replicates. *** = *P* < 0.001 (determined using parametric *t* test). (*D*) Cocultured cells were treated with AlexaFluor-647 tagged TGF-β (100 nM) for 52 h. Then, K562 receiver cells were analyzed by flow cytometry. T cells secreting G2 GELYTAC were tested. Control GELYTAC targets mCherry instead of TGF-β. The measured concentration for TGF-β (G2), TGF-β scFv, and mCherry control GELYTAC (G2) at 72 h are 3.2 ± 1.1 nM, 195 ± 41 pM, and 32 ± 4.3 nM (*SI Appendix*, Figs. S5*G* and S7 *C–F*). Errors bars represent the SD from three biological replicates. ** = *P* < 0.01 (determined using parametric *t* test). (*E*) Cocultured cells were treated with AlexaFluor-647 tagged IL6R (100 nM) for 9 h. Then, K562 receiver cells were analyzed by flow cytometry. T cells secreting G2 GELYTAC were tested. Control GELYTAC targets mCherry instead of IL6R. The measured concentration for IL6R GELYTAC (G2), IL6R nanobody, and mCherry control GELYTAC (G2) at 24 h are 2.5 ± 0.54 nM, 24 ± 2.3 nM, and 5.7 ± 1.3 nM (*SI Appendix*, Figs. S5*I* and S7 *G* and *H*). Errors bars represent the SD from three biological replicates. * = *P* < 0.1 (determined using parametric *t* test). (*F*) Experiment performed as detailed in (*E*) but measuring fold change in AlexaFluor-647 MFI in human primary T cells from the coculture system. Errors bars represent the SD from three biological replicates. *** = *P* < 0.001 (determined using parametric *t* test).

To potentially enable spatial specificity for GELYTACs, we imagined that GELYTAC could be integrated into adoptively transferred T cell therapy, such as CAR-T therapy. In adoptive T cell therapy, engineered primary T cells home to tumors and proliferate in the tumor microenvironment based on recognition of specific tumor antigens. Furthermore, primary T cells can be engineered to secrete factors under the control of logic gates determined by recognition of tumor-specific factors (44). Thus, secretion from T cells could enable spatial specificity at the tumor site. To demonstrate the feasibility of this approach, we transduced donor human primary T cells with retrovirus encoding mCherry G2 GELYTAC (Fig. 5*A*). We observed robust GELYTAC secretion (*SI Appendix*, Fig. S5 *A* and *B*), and upon coincubation of GELYTAC-secreting T cells with K562 cells in media supplemented with 100 nM mCherry, observed 5.25-fold and 2.2-fold increases in mCherry MFI in K562 and T cells, respectively (Fig. 5 *B* and *C*). No significant increase in mCherry MFI was observed in controls with IL6R GELYTAC or mCherry nanobody. Interestingly, while there were differences in secretion of mCherry GELYTACs across primary T cells from three different donors, we noticed that the degree of mCherry uptake in T cells between the three donors was similar. Since the primary T cells were engineered to secrete GELYTACs, the IGF2 receptors were likely already saturated by GELYTACs on the membrane, so uptake in engineered T cells was more desensitized to increases or decreases in secretion (*SI Appendix*, Fig. S5*C*).

Last, we tested TGF-β and IL6R GELYTACs in the primary T cell coculture system. For these experiments, we cocultured human primary T cells secreting GELYTACs with K562 tumor cells and incubated with 100 nM of AF-647 tagged target proteins. For TGF-β GELYTACs, we observed a 1.6-fold increase in TGF-β uptake into K562 cells (Fig. 5*D* and *SI Appendix*, Fig. S5 *D–G*). For IL6R GELYTACs, we observed 2.2-fold increase and 5.2-fold increase in IL6R uptake into K562s and primary T cells, respectively (Fig. 5 *E* and *F* and *SI Appendix*, Fig. S5 *H* and *I*). For both TGF-β and IL6R experiments, we observed no major increase in AF-647 fluorescence in the mCherry GELYTAC or binder-only controls.

These results from human primary T cells demonstrate the potential for secreted GELYTACs to work in multiple cell types. Additionally, the ability of sender cells to act on themselves creates the possibility of feedback regulation of GELYTAC secretion.

## Discussion

In this study, we developed a small (20 to 30 kDa), genetically encoded LYTAC that can be secreted by primary human T cells. While there are multiple approaches to engineering genetically encoded LYTACs, a major benefit of using IGF2 is that it is a human protein, which is likely to decrease its immunogenicity compared to nonhuman proteins or computationally (45, 46) designed proteins (47). Nevertheless, the human immune system is complex and there exist mechanisms by which GELYTACs could still elicit anti-drug antibodies (48). An advantage of GELYTACs is its modularity, so components such as the linker or nanobody that are more likely to be immunogenic can be humanized or swapped for alternatives to minimize immunogenicity (49). During our study, other work was published that utilized a protein-based targeting chimera composed of two computationally designed binders to internalize EGFR via cell secretion (21), which could be immunogenic. Other work was also published during the preparation of this manuscript that utilizes IGF2-binder fusions but does not contain cell therapy applications or directed evolution (22).

To improve the potency of GELYTACs as mediators of extracellular protein degradation, we evolved a mutant IGF2 that binds more strongly to IGF2R. Using directed evolution, we derived an IGF2 variant that, when integrated into a GELYTAC, was approximately 10-fold more potent in mediating mCherry internalization. An added benefit of the improved GELYTAC was its higher expression levels in the HEK293T/K562 coculture model and its lower propensity to aggregate. This suggests that the improved performance of the evolved GELYTAC not only stems from increased potency as a biologic but also from increased levels of active species.

We engineered primary T cells to secrete GELYTACs as a model for a future CAR-T therapy. CAR-T cells are designed to home to and proliferate within tumors and have been shown to deliver protein cargos efficiently to tumors in vivo (25, 27). Thus, we believe that CAR-T cells will be capable of delivering GELYTACs to the tumor microenvironment. The GELYTACs we report here have low molecular weights (20 to 30 kDa), and therefore likely short serum residence times (50). Therefore, we would expect GELYTACs that have diffused away from the targeted environment to be rapidly cleared from circulation. The concept of spatial selectivity by small biologics secreted by T cells has been demonstrated using bi-specific T cell engagers (24).

Finally, the GELYTAC platform synergizes with several recent technologies for therapeutic development. For example, computational methods for de novo protein design as illustrated by Baker and coworkers’ recent work (21) can be deployed to optimize GELYTACs or to add additional functionality. Last, because they are genetically encoded, GELYTACs can be delivered via other forms of genetic medicine such as mRNA and viral gene therapy vectors.

## Materials and Methods

### Recombinant GELYTACs Production.

BL21 DE3 *E. coli* (Agilent) were transformed with a vector containing sequences encoding for GELYTACs or nanobody only controls with pelB signal sequence for localization to the periplasm for disulfide bond formation. A colony was picked into 10 mL of LB (supplemented with 2% glucose and kanamycin) overnight at 37 °C. The next day, the starter culture was added to 1L of 2×YT (supplemented with antibiotics) and grown at 37 °C to an OD600 ~1.0 to 1.3. The culture was then induced at 1 mM and grown overnight at 16 °C at 225 rpm. The next day, the culture was centrifuged at 7,000 g for 10 min and the supernatant discarded. The cell pellet was then resuspended thoroughly with 20 mL 1× TES (0.2 M Tris, pH = 8, 0.5 mM EDTA, 0.5 M sucrose) and then the mixture was added to 20 mL of ice-cold ddH_2_O (supplemented with protease inhibitor). The mixture was then incubated overnight at 4 °C with shaking. The next day the mixture was centrifuged at 16,000 × g. The supernatant was then filtered using a 5 µM filter and then purified using Ni NTA column (Cytiva/GE Healthcare) on an FPLC (AKTA Pure). Following Ni-NTA purification, the mixture was then purified using size exclusion chromatography (Superdex 75 Increase 10/300 GL) and only the monomer was isolated.

For Figs. 2 *B* and *D* and 3 *A* and *F*, the GELYTACs were mixed with mCherry-containing media before cells (also in mCherry media) were added. For Fig. 2*C* and *SI Appendix*, Fig. S1 *B* and *C*, GELYTAC was added directly to cells in mCherry supplemented media. The mCherry GELYTACs dose–response curve shown in Fig. 2*B* is the same data as the mCherry GELYTACs (original) dose–response curve in Fig. 3*F*, and was set up alongside the mCherry GELYTACs (G2) does curve in Fig. 3*F*.

### Yeast Display.

The yeast culture and display protocols and library generation protocols are described in depth in *SI Appendix* and previously published studies (38).

### Cell Culture (Excluding Primary T Cell Culture).

All cell lines used were less than passage 20. HEK 293 T cells (ATCC) and 293GP retroviral packaging line [gift from Surgery Branch (National Cancer Institute, NIH)] were cultured in a DMEM (Gibco) supplemented with 10% fetal bovine serum (FBS), 1% Glutamax (Gibco), 100 units/mL penicillin, and 100 mg/mL streptomycin at 37 °C under 5% CO_2_. K562’s (ATCC) was cultured in RPMI (Sigma Aldrich) supplemented with 10% FBS, 1% Glutamax (Gibco), 100 units/mL penicillin, and 100 mg/mL streptomycin at 37 °C under 5% CO_2_.

### HEK293T Coculture with K562.

Twelve-well plates were coated with HFN (1 mL of HFN from Sigma added to 50 mL PBS), by adding 700 µL of HFN supplemented PBS to the wells. A 70 to 90% confluent HEK293T T75 flask was lifted, by aspirating media and adding ~1 to 2 mL trypsin. Cells were diluted to 3.5e5 cells/mL, and 1 mL was added to each well and slightly agitated before returning it in the incubator. On the next day, cells were transfected with plasmids encoding for GELYTACs or controls.

To make the transfection mix, 1,000 ng of plasmids encoding for GELYTACs or controls were added to 100 µL of blank DMEM. The mixture was mixed by flicking the tube, and then 5 µL of polyethylene imine (PEI). The tube was flicked gently to homogenize, and allowed to incubate for 20 min. After incubation, the transfection mix was added directly to cells dropwise, cells were returned to the incubator for 12 to 18 h. After 12 to 18 h, media + the transfection mix were aspirated and replated with 1 mL of RPMI mCherry with 3.0e5 GFP K562/mL. After 3 h 100 nM of soluble antigen (i.e., mCherry, TGF- β, or IL6R) was spiked into the coculture.

At the time of analysis, the GFP K562’s was analyzed by flow cytometry for median fluorescence of mCherry or AlexaFluor-647 tagged proteins. GFP was used to distinguish between K562 cells and HEK293T cells.

### Retrovirus Production.

Retroviral supernatant was packaged using 293GP cells and the RD114 envelope plasmid. In brief, 11 μg RD114 and 22 μg of the corresponding MSGV1 transfer plasmid that contains GELYTACs or controls were delivered to 293GP cells grown on 150 mm HFN dishes (Corning) to 80% confluency by transient transfection with Lipofectamine 2000 (Thermo Fisher). Media was replenished every 24 h. Virus production was performed side-by-side for comparable GELYTACs and control constructs. Retroviral supernatant was harvested 48-h post transfection. Supernatant from replicate dishes were pooled, centrifuged to deplete cell debris, and stored at −80 °C until use. A similar protocol is described in previously published studies (51, 52).

### T Cell Activation.

Anonymous healthy donor buffy coats were collected by and purchased from the Stanford Blood Center under an IRB-exempt protocol. Primary human T cells (CD3+) were isolated using the RosetteSep Human T cell Enrichment kit (Stem Cell Technologies) according to the manufacturer’s protocol using Lymphoprep density gradient medium and SepMate-50 tubes. All purified T cells were cryopreserved in CryoStor CS10 media (Stem Cell Technologies).

On Day 0, primary human T cells were thawed and activated with anti-CD3/CD28 Human T-Expander Dynabeads (Thermo Fisher) at a 3:1 bead to cell ratio. On Day 2 virus coated culture plates were prepared on non-TC-treated 12-well plates that had been pre-coated with RetroNectin (Takara Bio) according to the manufacturer’s instructions, by incubating with 1 mL of retroviral supernatant (2 × 10^7^ to 5 × 10^7^ TU/mL) and centrifugation at 3,200 rpm, 32 °C for 2 h. The supernatant was subsequently aspirated from the wells and 0.5 × 10^6^ T cells were added in 1 mL of T cell media comprised of: AIM V (Thermo Fisher), 5% fetal bovine serum (FBS), 100 U/mL penicillin (Gibco), 100 mg/mL streptomycin (Gibco), 2 mM L-glutamine (Gibco), 10 mM HEPES (Gibco), and 100 U/mL rhIL2 (Peprotech). After addition of the T cells, the plates were gently spun down at 1,200 rpm for 2 min then incubated for 24 h at 37 °C 5% CO_2_. This transduction process was repeated on Day 3. Dynabeads were removed on Day 4 by magnetic separation. Cells were maintained between 0.4 and 2 × 10^6^ cells/mL and expanded until Day 10. A similar protocol is described in previously published studies (51, 52).

### T Cell Coculture with K562 Cells.

Coculture experiments were conducted with primary T cells 7 to 14 d post retroviral transduction. For mCherry and IL6R GELYTACs, 0.6e6 T cells were cocultured with 0.3e6 K562’s. For TGF-β GELYTACs, 0.9e6 T cells were cocultured with 0.1e6 K562 cells. Coculture was set up with complete RPMI supplemented with 100 U/mL rhIL2.

At the time of analysis, the GFP K562’s and T cells were analyzed by flow cytometry for median fluorescence of mCherry or AlexaFluor-647 tagged proteins. GFP and/ or differences in forward and sider scatter were used to distinguish between K562 cells and T cells.

### Flow Cytometry.

First, 300 µL of K562 and or T cell culture was transferred to a 96-well V-bottom plate and spun down (500 g for 1 min). Cells were then washed 1× PBS w/0.5% supplemented bovine serum albumen (BSA). Cells were then incubated with SYTOX Blue (1:1,000 dilution) for 5 min. Flow cytometry was performed a BioRad ZE5 flow cytometer, and analysis was performed using the FlowJo software package. Gating was performed on single cells and live cells. This instrument is equipped with a 405-nm violet laser, a 488-nm blue laser, a 561-nm green laser, and a 639-nm red laser.

For Figs. 2 *B* and *D* and 4 *C* and *D*, K562 cells were washed with PBS and then incubated with 5% trypsin for 1 min at 37 °C. After trypsin treatment, trypsin was quenched with PBS supplemented with 0.5% BSA and then subsequently subjected to the standard flow protocol described above. This was done to cleave GELYTACs and receptors on the cell surface that may contribute to mCherry signal from noninternalized mCherry.

### Western Blot Protocols.

Conditioned media from transfected HEK293T cells or transduced T cells were collected (cells were removed by centrifugation) and LDS Sample Buffer (4×) (for reduced blot 4× loading buffer was supplemented with 10% 1 M DTT) and boiled at 95 °C for 10 min. The recommended volume (15 µL for 26-well gel or 30 µL for 18-well gel) was loaded on a SDS–PAGE (10% Bis-Tris gel) and then transferred to a nitrocellulose membrane. After transfer, the blot was blocked with Odyssey Blocking Buffer (PBS or TBS) (LI-COR) for 1 h at room temperature with gentle shaking. Membranes were stained with M2 anti-FLAG (Sigma Aldrich) for 1 h at room temperature or at 4 °C with gentle shaking, then washed three times with PBS-T for 5 min each. The membrane was then incubated with 800CW goat anti-mouse IgG (1:10,000) in Odyssey Blocking Buffer (PBS or TBS) for 1 h at room temperature with gentle shaking. Membranes were washed three times with TBS-T, and then imaged using an OdysseyCLxImager (LI-COR). Quantification of band intensities was performed using Image Studio Software (LI-COR). The blot of TGF-β GELYTAC secretion in *SI Appendix*, Fig. S1*D* is a crop of the blot of the TGF-β GELYTAC secretion time course shown in *SI Appendix*, Fig. S7*C*.

### Determining GELYTACs and Controls Concentration in Coculture Assay.

Conditioned media from transfected HEK293T cells or transduced T cells were collected and subjected to the western blot protocol described above. Alongside the supernatant samples were a series of standards (samples of recombinant FLAG-tagged GELYTACs at set concentration). A standard curve was determined by plotting signal of the recombinant GELYTAC band versus the mass of recombinant GELYTAC loaded.

To calculate the mass of the secreted GELYTACs loaded onto the gel, the intensity of the secreted GELYTAC band was divided by the slope of the standard curve. From calculated mass, the concentration can be calculated.

### Confocal Imagining.

K562 cells were plated on HFN coated glass coverslip bottom well plates with phenol red free complete RPMI. On the next day, cells were treated with 275 nM of recombinant mCherry GELYTAC or controls and 100 nM of mCherry for 1 h. The well plates were then imaged live using a Zeiss AxioObserver microscope. This microscope is equipped with 603 oil immersion objectives, outfitted with a Yokogawa spinning disk confocal head, Cascade II:512 camera, a Quad-band notch dichroic mirror (405/488/ 568/647), and 405 (diode), 491 (DPSS), 561 (DPSS) and 640 nm (diode) lasers (all 50 mW). DAPI (405 laser excitation, 445/40 emission), Alexa Fluor488 (491 laser excitation, 528/38 emission) and AlexaFluor647 (640 laser excitation, 700/75 emission), and differential interference contrast (DIC) images were acquired through a 60× oil-immersion lens. Acquisition times ranged from 100 to 2,000 ms. All images were collected and processed using SlideBook 6.0 software (Intelligent Imaging Innovations). A similar protocol is described in previously published studies (53).

### Knock-Out Cell Line Generation.

Knock-out K562 cell lines were generated by electroporating the sgRNA (Synthego) Cas9 (ID Technologies) complex using a Lonza 4D Nucleofector. The cell line was generated using Synthego’s protocols.

### Top–Down Protein Mass Spectrometry.

After protein expression and purification, the protein samples were further buffer exchanged using a methanol/chloroform/water precipitation and resolubilization method (54). Here, 300 μL of 10 mM TCEP prepared in cold LC/MS-grade water (4 °C) was added to 100 μL of protein solution. Then, 400 μL of cold methanol (−20 °C) was added to the protein solution and vortexed for 30 s followed by 100 μL of cold chloroform (−20 °C) and an additional 30 s of vortexing. The sample was centrifuged for 10 min at 18,000*g* at 4 °C after which a biphasic mixture was created with a protein pellet present at the interface. The top layer of the solution was discarded without disturbing the protein pellet. Then, 400 μL of cold methanol (−20 °C) was added to the sample and gently vortexed. The sample was centrifuged for 10 min at 18,000 *g* at 4 °C after which the supernatant was discarded. The cold methanol and centrifugation washing step was repeated two additional times. Protein pellets were resolubilized with 4 µL of 80% formic acid (−20 °C) and diluted to 1% formic acid with 80:20 water: acetonitrile. Top–down LC–MS was carried out using an Agilent 1260 Infinity II high-performance liquid chromatography (HPLC) system coupled to an Agilent 6230 ToF LC/MS (Agilent Technologies). Samples were injected onto an Agilent PLRP-S column (2.1 × 50 mm, 5 µm particle size, 1,000 Å pore size) using a gradient of 10 to 90% mobile phase B (0 to 5 min, 10% B; 5 to 15 min, 20 to 65% B; 15 to 18 min, 65 to 90% B, 18 to 22 min, 90% B; 22 to 25 min, 10% B; mobile phase A set to 0.2% formic acid in water; mobile phase B set to 0.2% formic acid in acetonitrile). Flow rate was set to 200 μL/min with a column temperature of 60 °C. Mass spectra were taken at a scan rate of 1 Hz over a 200 to 3,200 *m/z* scan range with the ToF set to extended dynamic range. The mass spectrometer was operated using a dual Agilent jet stream (AJS) high-sensitivity ion source with the following instrument parameters: gas temperature (275 °C), drying gas (12 L/min), nebulizer (40 psi), sheath gas temperature (400 °C), sheath gas flow (12 L/min), VCap(3,000 V), nozzle voltage (2,000 V), fragmentor (250 V), skimmer (65 V), and Oct 1 RF Vpp (750 V). Mass spectra were output from the MassHunter (Agilent Technologies) software and analyzed using MASH Native (55) and UniDec (56). A similar protocol is described in previously published studies (57). Source mass spectrometry data are available via the MassIVE repository with identifier MSV000094184 and the PRIDE repository via ProteomeXchange with identifier PXD050196 (58, 59).

## Supplementary Material

Appendix 01 (PDF)

## Data Availability

All study data are included in the article and/or *SI Appendix*.

## References

[1] M. Békés, D. R. Langley, C. M. Crews, PROTAC targeted protein degraders: Past is prologue. Nat. Rev. Drug Discov. **21**, 181–200 (2022). 35042991 10.1038/s41573-021-00371-6 PMC8765495

[2] S. M. Banik , Lysosome-targeting chimaeras for degradation of extracellular proteins. Nature **584**, 291–297 (2020). 32728216 10.1038/s41586-020-2545-9 PMC7727926

[3] G. Ahn , LYTACs that engage the asialoglycoprotein receptor for targeted protein degradation. Nat. Chem. Biol. **17**, 937–946 (2021). 33767387 10.1038/s41589-021-00770-1 PMC8387313

[4] G. Ahn , Elucidating the cellular determinants of targeted membrane protein degradation by lysosome-targeting chimeras. Science. **382**, 281 (2023). 10.1126/science.adf6249 PMC10766146 37856615

[5] A. D. Cotton, D. P. Nguyen, J. A. Gramespacher, I. B. Seiple, J. A. Wells, Development of antibody-based PROTACs for the degradation of the cell-surface immune checkpoint protein PD-l1. J. Am. Chem. Soc. **143**, 593–598 (2021). 33395526 10.1021/jacs.0c10008 PMC8154509

[6] H. Marei , Antibody targeting of E3 ubiquitin ligases for receptor degradation. Nature **610**, 182–189 (2022). 36131013 10.1038/s41586-022-05235-6 PMC9534761

[7] K. Pance , Modular cytokine receptor-targeting chimeras for targeted degradation of cell surface and extracellular proteins. Nat. Biotechnol. **41**, 273–281 (2023). 36138170 10.1038/s41587-022-01456-2 PMC9931583

[8] D. F. Caianiello , Bifunctional small molecules that mediate the degradation of extracellular proteins. Nat. Chem. Biol. **17**, 947–953 (2021). 34413525 10.1038/s41589-021-00851-1

[9] H. Zhang , Covalently engineered nanobody chimeras for targeted membrane protein degradation. J. Am. Chem. Soc. **143**, 16377–16382 (2021). 34596400 10.1021/jacs.1c08521

[10] Y. Miao , Han bispecific aptamer chimeras enable targeted protein degradation on cell membranes. Angew. Chem. Int. Ed. **60**, 11267–11271 (2021). 10.1002/anie.202102170 33634555

[11] J. Zheng , Bifunctional compounds as molecular degraders for integrin-facilitated targeted protein degradation. J. Am. Chem. Soc. **144**, 21831–21836 (2022). 36417563 10.1021/jacs.2c08367

[12] R. Sun, Z. Meng, H. Lee, R. Offringa, C. Niehrs, ROTACs leverage signaling-incompetent R-spondin for targeted protein degradation. Cell Chem. Biol. **30**, 739–752 (2023). 37321224 10.1016/j.chembiol.2023.05.010

[13] E. Loppinet , Targeted lysosomal degradation of secreted and cell surface proteins through the LRP-1 pathway. J. Am. Chem. Soc. **145**, 18705–18710 (2023). 37590164 10.1021/jacs.3c05109 PMC10809789

[14] J. Duque-Jimenez , Transferrin Receptor Targeting Chimeras (TransTACs) for membrane protein degradation. bioRxiv [Preprint] (2023), 10.1101/2023.08.10.552782 (Accessed 14 August 2023). PMC11839386 39322661

[15] D. H. Siepe, L. K. Picton, K. C. Garcia, Receptor elimination by E3 ubiquitin ligase recruitment (REULR): A targeted protein degradation toolbox. ACS. Synth. Biol. **12**, 1081–1093 (2023). 10.1021/acssynbio.2c00587 PMC10127277 37011906

[16] Y. Zhou, P. Teng, N. T. Montgomery, X. Li, W. Tang, Development of triantennary N-Acetylgalactosamine conjugates as degraders for extracellular proteins. ACS Cent. Sci. **7**, 499–506 (2021). 33791431 10.1021/acscentsci.1c00146 PMC8006166

[17] X. Zhang , Site-specific chemoenzymatic conjugation of high-affinity M6P glycan ligands to antibodies for targeted protein degradation. ACS Chem. Biol. **17**, 3013–3023 (2022). 35316032 10.1021/acschembio.1c00751 PMC9492806

[18] Y. Wu , Aptamer-LYTACs for targeted degradation of extracellular and membrane proteins. Angew. Chem. Int. Ed. **62** (2023). 10.1002/anie.202218106 36722696

[19] K. Hamada , Development of a bispecific DNA-aptamer-based lysosome-targeting chimera for HER2 protein degradation. Cell Rep. Phys. Sci. **4**, 101296 (2023).

[20] R. A. Howell, R. Wang, D. M. Mcdonald, D. A. Spiegel, Bifunctional molecules that induce targeted degradation and transcytosis of extracellular proteins in brain cells. ChemRxiv [Preprint] (2023). 10.26434/chemrxiv-2023-5l14b-v2 (Accessed 22 September 2023). 38855935

[21] B. Huang , Designed endocytosis-triggering proteins mediate targeted degradation. bioRxiv [Preprint] (2023). 10.1101/2023.08.19.55332 (Accessed 18 September 2023).

[22] B. Zhang , Insulin-like growth factor 2 (IGF2)-fused lysosomal targeting chimeras for degradation of extracellular and membrane proteins. J. Am. Chem. Soc. **145**, 24272–24283 (2023). 37899626 10.1021/jacs.3c08886

[23] L. Labanieh, C. L. Mackall, CAR immune cells: Design principles, resistance and the next generation. Nature **614**, 635–648 (2023). 36813894 10.1038/s41586-023-05707-3

[24] S. Rafiq , Targeted delivery of a PD-1-blocking scFV by CAR-T cells enhances anti-tumor efficacy in vivo. Nat. Biotechnol. **36**, 847–858 (2018). 30102295 10.1038/nbt.4195 PMC6126939

[25] B. D. Choi , CAR-T cells secreting BiTEs circumvent antigen escape without detectable toxicity. Nat. Biotechnol. **37**, 1049–1058 (2019). 31332324 10.1038/s41587-019-0192-1

[26] Y. Yin , Locally secreted BiTEs complement CAR T cells by enhancing killing of antigen heterogeneous solid tumors. Mol. Ther. **30**, 2537–2553 (2022). 35570396 10.1016/j.ymthe.2022.05.011 PMC9263323

[27] W. Xia , Engineering a HER2-CAR-NK cell secreting soluble programmed cell death protein with superior antitumor efficacy. Int. J. Mol. Sci. **24**, 1–17 (2023). 10.3390/ijms24076843 PMC10094803 37047817

[28] K. J. Curran , Enhancing antitumor efficacy of chimeric antigen receptor T cells through constitutive CD40L expression. Mol. Ther. **23**, 769–778 (2015). 25582824 10.1038/mt.2015.4 PMC4395796

[29] O. O. Yeku, T. J. Purdon, M. Koneru, D. Spriggs, R. J. Brentjens, Armored CAR T cells enhance antitumor efficacy and overcome the tumor microenvironment. Sci. Rep. **7**, 1–14 (2017). 28874817 10.1038/s41598-017-10940-8 PMC5585170

[30] H. J. Pegram , Tumor-targeted T cells modified to secrete IL-12 eradicate systemic tumors without need for prior conditioning. Blood **119**, 4133–4141 (2012). 22354001 10.1182/blood-2011-12-400044 PMC3359735

[31] J. Brown , Structure and functional analysis of the IGF-II/IGF2R interaction. EMBO **27**, 265–276 (2008). 10.1038/sj.emboj.7601938 PMC2206120 18046459

[32] Y. Oka, L. M. Rozek, M. P. Czech, Direct demonstration of rapid insulin-like growth factor II receptor internalization and recycling in rat adipocytes. Insulin stimulates 125I-insulin-like growth factor II degradation by modulating the IGF-II receptor recycling process. JBC **260**, 9435–9442 (1985). 2991246

[33] M. E. Zavorka, C. M. Connelly, R. Grosely, G. Richard McDonald, Inhibition of insulin-like growth factor II (IGF-II) -dependent cell growth by multidentate pentamannosyl 6-phosphate-based ligands targeting the mannose 6-phosphate/IGF-II receptor. Oncotarget **7**, 10–16 (2016). 10.18632/oncotarget.11493 PMC5308735 27694692

[34] P. C. Fridy , A robust pipeline for rapid production of versatilenanobody repertoires. Nat. Methods **11**, 1253–1260 (2014). 25362362 10.1038/nmeth.3170 PMC4272012

[35] L. Huang, D. Pike, D. E. Sleat, V. Nanda, P. Lobel, Potential pitfalls and solutions for use of fluorescent fusion proteins to study the lysosome. PLoS One **9**, e88893 (2014). 24586430 10.1371/journal.pone.0088893 PMC3931630

[36] C. Delaine , A novel binding site for the human insulin-like growth factor-II (IGF-II)/mannose 6-phosphate receptor on IGF-II. JBC **282**, 18886–18894 (2007). 10.1074/jbc.M700531200 17475626

[37] S. S. Lam , Directed evolution of APEX2 for electron microscopy and proximity labeling. Nat. Methods **12**, 51–54 (2014). 25419960 10.1038/nmeth.3179 PMC4296904

[38] T. C. Branon , Efficient proximity labeling in living cells and organisms with TurboID. Nat. Biotechnol. **36**, 880–898 (2018). 30125270 10.1038/nbt.4201 PMC6126969

[39] J. D. Martell , A split horseradish peroxidase for the detection of intercellular protein-protein interactions and sensitive visualization of synapses. Nat. Biotechnol. **34**, 774–780 (2016). 27240195 10.1038/nbt.3563 PMC4942342

[40] S. Y. Lee , Engineered allostery in light-regulated LOV-Turbo enables precise spatiotemporal control of proximity labeling in living cells. Nat. Methods **20**, 908–917 (2023). 37188954 10.1038/s41592-023-01880-5 PMC10539039

[41] A. Moulin , Structures of a pan-specific antagonist antibody complexed to different isoforms of TGFβ reveal structural plasticity of antibody-antigen interactions. Protein Sci. **23**, 1698–1707 (2014). 25209176 10.1002/pro.2548 PMC4253810

[42] M. Van Roy , The preclinical pharmacology of the high affinity anti-IL-6R Nanobody® ALX-0061 supports its clinical development in rheumatoid arthritis. Arthritis Res. Ther. **17**, 135 (2015). 25994180 10.1186/s13075-015-0651-0 PMC4476083

[43] C. K. Ala, A. L. Klein, J. J. Moslehi, Cancer treatment-associated pericardial disease: Epidemiology, clinical presentation, diagnosis, and management. Curr. Cardiol. Rep. **21**, 1–9 (2019). 31768769 10.1007/s11886-019-1225-6

[44] K. T. Roybal , Engineering T cells with customized therapeutic response programs using synthetic notch receptors. Cell **167**, 419–432 (2016). 27693353 10.1016/j.cell.2016.09.011 PMC5072533

[45] A. S. De Groot, D. W. Scott, Immunogenicity of protein therapeutics. Trends Immunol. **28**, 482–490 (2007). 17964218 10.1016/j.it.2007.07.011

[46] H. Schellekens, Bioequivalence and the immunogenicity of biopharmaceuticals. Nat. Rev. Drug Discov. **1**, 457–462 (2002). 12119747 10.1038/nrd818

[47] H. Lu, Z. Cheng, Y. Hu, L. V. Tang, What can de novo protein design bring to the treatment of hematological disorders? Biology **12**, 166 (2023). 36829445 10.3390/biology12020166 PMC9952452

[48] R. Mosch, H. J. Guchelaar, Immunogenicity of monoclonal antibodies and the potential use of HLA haplotypes to predict vulnerable patients. Front. Immunol. **13**, 1–11 (2022). 10.3389/fimmu.2022.885672 PMC9249215 35784343

[49] C. Vincke , General strategy to humanize a camelid single-domain antibody and identification of a universal humanized nanobody scaffold. J. Biol. Chem. **284**, 3273–3284 (2009). 19010777 10.1074/jbc.M806889200

[50] D. K. Shah, Pharmacokinetic and pharmacodynamic considerations for the next generation protein therapeutics. J. Pharmacokinet. Pharmacodyn **42**, 553–571 (2015). 26373957 10.1007/s10928-015-9447-8 PMC4593704

[51] L. Labanieh , Enhanced safety and efficacy of protease-regulated CAR-T cell receptors. Cell **185**, 1745–1763.e22 (2022). 35483375 10.1016/j.cell.2022.03.041 PMC9467936

[52] S. A. Yamada-Hunter , Engineered CD47 protects T cells for enhanced antitumor immunity. bioRxiv [Preprint] (2023). 10.1101/2023.06.20.545790 (Accessed 31 January 2024). PMC11168929 38750365

[53] W. Qin , Dynamic mapping of proteome trafficking within and between living cells by TransitID. Cell **186**, 3307–3324.e30 (2023). 37385249 10.1016/j.cell.2023.05.044 PMC10527209

[54] H. T. Rogers , Comprehensive characterization of endogenous phospholamban proteoforms enabled by photocleavable surfactant and top-down proteomics. Anal. Chem. **95**, 13091–13100 (2023). 37607050 10.1021/acs.analchem.3c01618 PMC10597709

[55] E. J. Larson , MASH Native: A unified solution for native top-down proteomics data processing. Bioinformatics **39**, btad359 (2023). 37294807 10.1093/bioinformatics/btad359 PMC10283151

[56] M. T. Marty , Bayesian deconvolution of mass and ion mobility spectra: From binary interactions to polydisperse ensembles. Anal. Chem. **87**, 4370–4376 (2015). 25799115 10.1021/acs.analchem.5b00140 PMC4594776

[57] J. Donnelly , A genome-wide CRISPR screen identifies sortilin as the receptor responsible for Galectin-1 lysosomal trafficking. bioRxiv [Preprint] (2024). 10.1101/2024.01.03.574113 (Accessed 31 January 2024).

[58] J. L. Yang , Directed Evolution of Genetically Encoded LYTACs for Cell-Mediated Deliver. MassIVe. MSV000094184. https://massive.ucsd.edu/ProteoSAFe/private-dataset.jsp?task=5b9f5171304d48de888c9f446e908bb9. Deposited 27 February 2024. 10.1073/pnas.2320053121 PMC10990137 38513100

[59] J. L. Yang , Directed Evolution of Genetically Encoded LYTACs for Cell-Mediated Deliver. PRIDE repository via ProteomeXchange. PXD050196. https://proteomecentral.proteomexchange.org/cgi/GetDataset?ID=PXD050196. Deposited 27 February 2024. 10.1073/pnas.2320053121 PMC10990137 38513100

